# Healing of Periodontal Suprabony Defects following Treatment with Open Flap Debridement with or without Hyaluronic Acid (HA) Application

**DOI:** 10.3390/medicina60050829

**Published:** 2024-05-17

**Authors:** Octavia Carolina Vela, Marius Boariu, Darian Rusu, Vincenzo Iorio-Siciliano, Luca Ramaglia, Simina Boia, Viorelia Radulescu, Ioana Ilyes, Stefan-Ioan Stratul

**Affiliations:** 1 Department of Periodontology, Faculty of Dental Medicine, Anton Sculean Research Center for Periodontal and Peri-Implant Diseases, Victor Babes University of Medicine and Pharmacy, 300041 Timisoara, Romania; vela.octavia@umft.ro (O.C.V.); rusu.darian@umft.ro (D.R.); simina.boia@umft.ro (S.B.); viorelia.radulescu@umft.ro (V.R.); ioana.veja@umft.ro (I.I.); stratul.stefan@umft.ro (S.-I.S.); 2 Department of Endodontics, Faculty of Dental Medicine, TADERP Research Center, Victor Babes University of Medicine and Pharmacy, 300041 Timisoara, Romania; 3 Department of Periodontology, School of Dental Medicine, University of Naples Federico II, 80138 Naples, Italy; enzois@libero.it (V.I.-S.); luca.ramaglia@unina.it (L.R.)

**Keywords:** open flap debridement, suprabony defects, hyaluronic acid, periodontal pocket, periodontal regeneration

## Abstract

*Background and Objectives:* This randomized, double-arm, multicentric clinical trial aims to compare the clinical outcomes following the treatment of suprabony periodontal defects using open flap debridement (OFD) with or without the application of hyaluronic acid (HA). *Materials and Methods:* Sixty systemically healthy patients with at least two teeth presenting suprabony periodontal defects were randomly assigned with a 1:1 allocation ratio using computer-generated tables into a test (OFD + HA) or control group (OFD). The main outcome variable was clinical attachment level (CAL). The secondary outcome variables were changes in mean probing pocket depth (PPD), gingival recession (GR), full-mouth plaque score (FMPS), and full-mouth bleeding score (FMBS). All clinical measurements were carried out at baseline and 12 months. *Results:* Sixty patients, thirty in each group, were available for statistical analysis. The mean CAL gain was statistically significantly different (*p* < 0.001) in the test group compared with the control group (3.06 ± 1.13 mm vs. 1.44 ± 1.07 mm). PPD reduction of test group measurements (3.28 ± 1.14 mm) versus the control group measurements (2.61 ± 1.22 mm) were statistically significant (*p* = 0.032). GR changes were statistically significant only in the test group 0.74 ± 1.03 mm (*p* < 0.001). FMBS and FMPS revealed a statistically significant improvement mostly in the test group. *Conclusions:* Suprabony periodontal defects could benefit from the additional application of HA in conjunction with OFD in terms of improvement of the clinical parameters compared with OFD alone.

## 1. Introduction

The main goal of periodontal therapy is to treat the infection caused by periodontal pathogenic biofilm and to arrest or slow down further attachment and bone loss, thus preventing further tooth loss [1]. Clinically, successful treatment is defined by reductions in probing pocket depth (PPD) and bleeding score (BOP), along with gains in clinical attachment level (CAL) and radiographic bone [2]. There is ample evidence that, in the great majority of cases, these goals can be achieved with the first and second steps of periodontal treatment. These consist of patient motivation and instructions for the successful removal of supragingival dental biofilm and the control of risk factors known to be associated with the deterioration of periodontal status, such as smoking and diabetes (step one), followed by nonsurgical subgingival instrumentation (step two) [3].

Regeneration of the supporting tissues lost due to periodontal disease refers to the reproduction or reconstitution of a lost or injured part, in contrast to repair, which describes healing by tissues that do not fully restore the architecture or the function of the lost part [4]. Although regeneration is an unpredictable goal, especially when clinical attachment loss reaches deeper structures, periodontal repair can still be achieved through surgical periodontal therapy [4]. 

Resective surgery is clinically efficient and leads predictably to pocket reduction. However, it results in a substantial increase in gingival recessions, dental hypersensitivity, and impaired aesthetics [1]. Due to their unfavorable anatomy, suprabony periodontal defects represent the least predictable defect type for regenerative periodontal therapy and are still a challenge for clinicians [5], frequently being treated using access flap surgery with or without osseous resection [1]. A recent study indicates that, in suprabony defects, the application of EMD in conjunction with OFD additionally improves clinical outcomes compared with OFD only in terms of clinical attachment level gain and probing depth reduction [6]. However, the search for new biomaterials that are user-friendly and do not require specific conditions to be successfully used continues, and hyaluronic acid stands as a biomaterial that holds the potential to fulfill these requirements. In recent years, increasing evidence has indicated that the use of hyaluronic acid (HA) in nonsurgical and surgical periodontal therapy provides a clinical improvement in terms of CAL gain and PPD reduction [7,8,9]. 

Hyaluronic acid (HA) is a naturally occurring non-sulfated glycosaminoglycan with a high molecular weight of 4000–20,000,000 Da [10]. The structure of HA consists of polyanionic disaccharide units of glucuronic acid and N-acetyl glucosamine connected by alternating bl-3 and bl-4 bonds [11]. It is a linear polysaccharide of the extracellular matrix of connective tissue, synovial fluid, embryonic mesenchyme, vitreous humor, skin, and many other organs and tissues of the body [11]. It can play a regulatory role in inflammatory response: the high-molecular-weight HA synthesized by hyaluronan synthase enzymes in the periodontal tissues, gingiva, periodontal ligament, and alveolar bone [12] undergoes extensive degradation to lower-molecular-weight molecules in chronically inflamed tissue, such as gingival tissue inflammation or in the postoperative period after implant or sinus lift surgery [13].

HA is considered an optimal biomaterial for tissue engineering, given its broad expression in connective tissue as well as the significant role it plays during organogenesis, cell migration, and development in general [14,15]. Non-crosslinked HA is biodegradable, biocompatible and bioresorbable. It is also well known to improve tissue lubrication in cartilage, guide cell growth and differentiation, and speed the healing and repair of chronic wounds [16]. Cross-linked HA has also been utilized for tissue engineering as a scaffold to further improve the overall mechanical performance and rigidity of the scaffolding material, supporting the growth of various cells [17,18].

HA has also been hypothesized to have influences on periodontal regeneration [19] because it is an essential component of the periodontal ligament matrix and has been shown to play various important roles in cell adhesion, migration, and differentiation, mediated by various HA-binding proteins and cell surface receptors such as CD44 [20]. Other advantages of HA include its anti-inflammatory activity and promotion of soft and hard tissue healing response, which may be of significant interest during periodontal regeneration [10]. HA has already been tested in patients with chronic periodontitis, with several clinical studies reporting the beneficial effects of HA on reducing the bleeding of probing scores and probing depths [21,22,23,24]. 

The results from a recent study demonstrated that HA increases the periodontal ligament cell numbers on dentin discs [25]. Likewise, another study reported that HA maintains high PDL cell viability and increases their proliferation and early osteogenic differentiation [26]. In periodontal surgery, HA was tested in the root coverage procedures [22,27,28] and in the treatment of intrabony defects combined or not with bone grafts [29,30] with encouraging results. So far, there is no literature evidence on the use of HA only in combination with (OFD) in the treatment of periodontal suprabony (horizontal) defects. In the context of the aforementioned literature, this randomized clinical trial is the first to present novel findings about the adjunctive effects of HA application in periodontal surgical treatment and aims to compare the clinical outcomes following treatment of suprabony osseous defects using OFD with or without the application of HA. The null hypothesis (H0) considered that no statistically significant difference in the mean change of CAL following periodontal surgical therapy with the adjunctive use of HA would be detected compared with OFD alone.

## 2. Materials and Methods

### 2.1. Study Design

**Figure 1:**
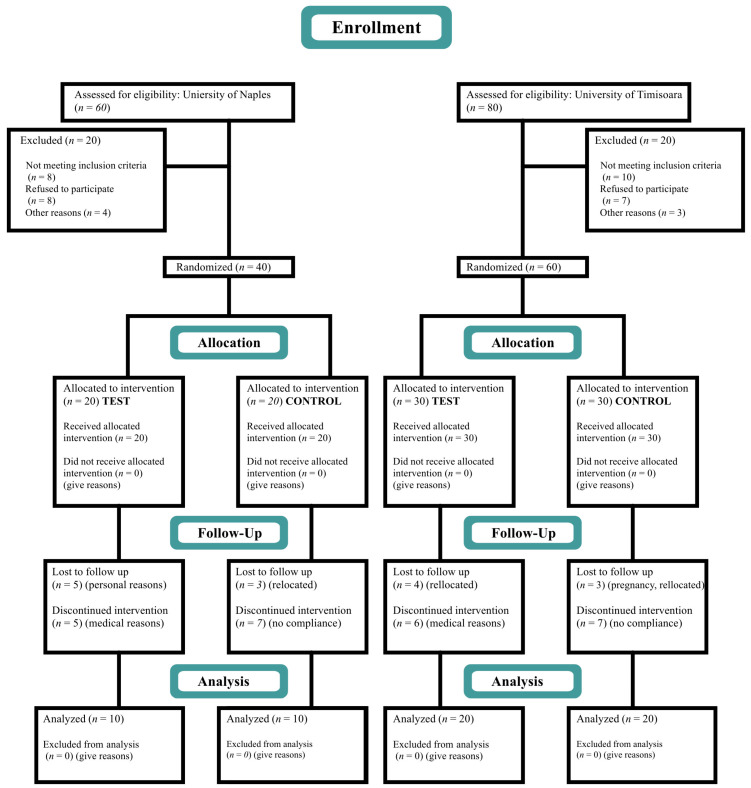
CONSORT diagram.

The study was designed as a double-arm, double-blind, randomized, controlled clinical trial. The suprabony defects of subjects allocated in the test group were treated with OFD and a cross-linked HA gel (hyaDENT BG, Bioscience, Dümmer, Germany) application, while the suprabony defects of the control group were treated with OFD alone. The study protocol was approved by the Scientific Research Ethics Committee of Victor Babes University of Medicine and Pharmacy, Timisoara, (Nr. Av 16/29 March 2021) and by the Ethical Committee of Azienda Ospedaliera Universitaria Federico II integrate con il Servizio Sanitare Regionale (nr. 235/21, registro DS486). The study was conducted between April 2021 and December 2023 and was registered in the ClinicalTrials.Gov Registry of Clinical Trials (NCT05073575). The protocol was performed in accordance with the Good Clinical Practice (GCPs) guidelines (1996) and the Declaration of Helsinki of 1975, as revised in 2013. The study was conducted using the current standards of clinical research outlined in the CONSORT guidelines (http://www.consort-statement.org, accessed on 1 February 2024). The CONSORT diagram is depicted in Figure 1.

### 2.2. Patient Selection

All patients receiving periodontal treatment at the Department of Periodontology, Faculty of Dentistry, Victor Babes University of Medicine and Pharmacy, Timisoara, Romania, and the Department of Periodontology, University of Naples Federico II, Italy, were screened for this study. A total of 60 nonsmoking, systemically healthy patients suffering from periodontitis stages II and III, grades A and B, and each presenting at least two suprabony defects participated in this trial between April 2021 and December 2022. Data were collected in both academic centers.

*Inclusion criteria:*

- Patients diagnosed with periodontitis stages III and IV, grade A/B [31].
- Age ≥ 18 years old.
- Single-rooted and multi-rooted teeth.
- Patients that did not meet the therapy targets at re-evaluation after completion of step 2 of periodontal therapy with respect to the presence of suprabony periodontal defects (i.e., defects displaying a predominantly horizontal pattern of bone loss) at a minimum of two adjacent teeth and a maximum of seven adjacent teeth in either the maxilla or the mandible, with a PPD ≥ 5 mm.
- Intrabony defect with an intraosseous component < 2 mm.

*Exclusion criteria*:

- Patients with systemic diseases.
- Prolonged antibiotic or anti-inflammatory treatment within 4 weeks before surgery.
- Pregnant or lactating.
- Tobacco smokers (≥10 cigarettes per day).
- Patients with a mean full-mouth plaque score (FMPS) [32] and full-mouth bleeding score (FMBS) [33] ≥ 25% after completion of step 2 therapy.
- Multi-rooted teeth with furcation involvement [34].
- Third molars or severely mispositioned teeth.
- Increased tooth mobility (grade II and III) [35].

### 2.3. Sample Size Calculation

Based on similar studies, a total of 26 patients in each group was needed to reject the null hypothesis [6,36]. Thus, to estimate the sample size for our study comparing the efficacy of OFD and HA versus OFD only on CAL gain as the main outcome variable, we anticipated an average difference in CAL changes of 3 mm between the two treatment groups. Assuming a standard deviation of 1.5 mm based on similar previous studies, a significance level (alpha) of 0.05 for a two-tailed test, and aiming for a study power of 80% to detect this difference if it truly exists, we conducted a sample size calculation. Using these parameters, our calculation suggested that a total of 60 samples (30 per group) would provide the necessary statistical power to detect the anticipated difference in CAL changes between the two groups, thus minimizing the risk of Type II errors. To compensate for patient dropouts during the study, a total of 100 subjects were enrolled. Of those, 40 (20 test and 20 control) were recruited in the Department of Periodontology, University of Naples Federico II, and 60 in the Department of Periodontology, Victor Babes University of Medicine and Pharmacy, Timisoara, (30 test and 30 control).

#### Randomization

The patients were randomly assigned to one of the two experimental procedures with a 1:1 allocation ratio through simple randomization, using a computerized random number generator (www.randomization.com, accessed on 1 April 2021). The allocation concealment was performed using numbers associated with the test (OFD + HA) or control (OFD alone) procedure. Prior to the procedure, the associated numbers were enclosed in opaque envelopes with the patients’ names on them. Thus, treatment assignment was kept secret. Treatment allocation was performed at the time of surgery after debridement of the suprabony defects by opening the envelope containing the number.

### 2.4. Blinding and Calibration

Treatment procedures were provided by an experienced periodontist (V.I-S) for the Department of Periodontology, University of Naples Federico II, and by another experienced periodontist (O.C.V) for the Department of Periodontology, Victor Babes University of Medicine and Pharmacy, Timisoara. All parameters were recorded at baseline and after 12 months by two calibrated and masked examiners (VR and LR). Examiners attended a training and calibration session on a total of 20 patients (kappa coefficient = 0.87).

### 2.5. Clinical Measurements

The following clinical parameters were recorded: PPD, defined as the distance from the gingival margin to the bottom of the pocket, and GR, defined as the distance from the gingival margin to the cemento-enamel junction (CEJ), both recorded to the nearest millimeter; CAL, defined as the distance from the CEJ to the bottom of the pocket and calculated as the sum of PPD and GR at sites with recession and PPD minus GR at sites without recession; full-mouth plaque score (FMPS), defined as a percentage of tooth sites revealing the presence of plaque [32]; and full-mouth bleeding score (FMBS), defined as a percentage of tooth sites with bleeding on probing (BOP) and recorded as the percentage of total surfaces (six aspects per tooth) [33]. The above-mentioned parameters were recorded using the same type of periodontal probe (PCPUNC-157, Hu-Friedy, Chicago, IL, USA) in both centers, at six points per tooth (mesio-buccal, mid-buccal, disto-buccal, mesio-oral, mid-oral, and disto-oral) 1 week before the surgical procedure and 12 months after. Only measurements from the area of interest, where the surgical procedure was performed, were used for statistical analysis.

### 2.6. Periodontal Therapy

After periodontal diagnosis was established according to the new classification system for periodontal diseases and conditions [31], all patients underwent steps 1 and 2 of periodontal therapy [3]. Step 1 of therapy included supragingival dental biofilm control; motivation; oral hygiene instructions (OHI); adjunctive therapies for gingival inflammation; and professional mechanical plaque removal (PMPR), which includes professional interventions aimed at removing supragingival plaque and calculus as well as possible plaque-retentive factors that impair oral hygiene practices. Step 2 of therapy (subgingival instrumentation) was performed according to the full-mouth disinfection protocol using ultrasonic (Acteon Group, Merignac, France) and hand instrumentation (Hu-Friedy, Chicago, IL, USA) under local anesthesia and was completed using air polishing (PROPHYflex 3, KaVo KERR, West Collins Orange, CA, USA) [37]. Afterward, OHI were reinforced. The patients were scheduled for a follow-up appointment 6 weeks after step 2 of therapy. The periodontal re-evaluation was conducted to assess if therapy goals were achieved and to verify the eligibility for participating in the study.

### 2.7. Surgical Procedure

**Figure 2:**
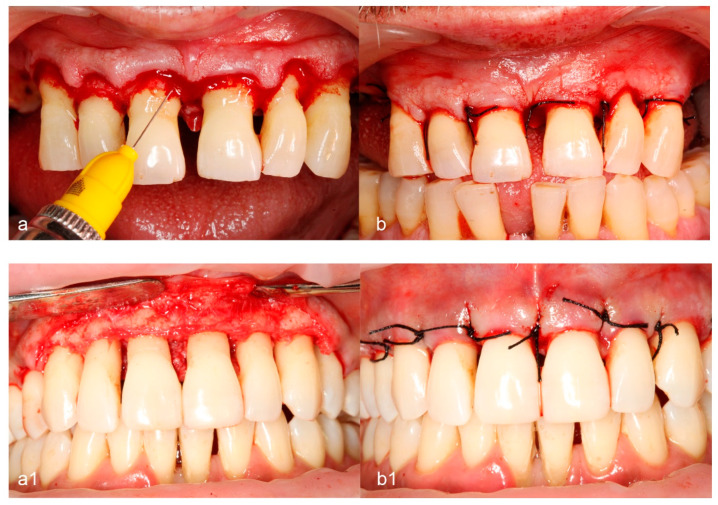
Illustration of test (**a**,**b**) and control (**a1**,**b1**) group cases: (**a**) intraoperative view of the defect with Hyadent (HA) application, (**b**) flap after suturing, (**a1**) intraoperative view of the defect, (**b1**) flap after suturing.

After local anesthesia (4% Ubistesin Forte, 3M ESPE AG, Seefeld, Germany), an access flap was prepared in both groups using identical techniques. Incisions were performed using 12D and 15C scalpel blades (Hu Friedy, Chicago, IL, USA) extending one tooth mesial or distal to the treated site to allow proper visualization and instrumentation. After the mucoperiosteal flap elevation, granulation tissue was removed using Gracey curettes (Hu-Friedy^®^, Chicago, IL, USA) and root instrumentation was performed with Gracey curettes and power-driven instruments (ultrasonic scaler, EMS^®^, Nyon, Switzerland). No modification of the bone contour was made. Once the surgical site preparation had ended, the patients were randomly assigned to one of the two experimental procedures by opening a sealed envelope. In the control group, no additional steps were taken, while in the test group, additional application of HA was performed. HA was applied according to the manufacturer’s instructions using an Uniject syringe (Hu-Friedy^®^ Anaesthetic Aspirating Syringe, 1.8cc, Type CW, 1/pk) with a disposable 30 g, 0.3 × 12 mm needle (Sopira^®^-Heraeus Kulzer, Hannau, Germany). A tension-free primary closure of the interdental papillae and the mucoperiosteal flaps was achieved after slightly dissecting the periosteum at the base of the pocket. Flaps were repositioned and sutured at the presurgical level using 4–0 black silk non-resorbable suturing material (18”, C-3 Needle 13 mm, 3/8 Circle Premium Reverse Cut, PermaSharp, Hu Friedy, Chicago, IL, USA). Post-surgical instructions were delivered, and pain and edema were controlled with 600 mg ibuprofen immediately before the surgical intervention and 4 h after. Control and test group representative images from the surgical therapy phase are shown in Figure 2.

### 2.8. Postoperative Care, Follow-Up, and Re-Evaluation

Subjects were instructed to rinse twice daily with 0.20% chlorhexidine digluconate (Dentaton Intensivo, Dental Greenline, GHIMAS^®^, Bologna, Italy) for the first 2 weeks, and self-performed oral hygiene procedures were interrupted only in the operated area so as not to tear the surgical stitches. In the rest of the oral cavity, the usual hygiene procedures were applied. Self-performed modified oral hygiene procedures, such as gentle brushing with a softheaded mechanical or manual toothbrush and no use of dental floss in the treated areas, were recommended for the next 2 weeks postoperative. No systemic antibiotics were prescribed. After 4 weeks, subjects were instructed to resume regular self-performed oral hygiene procedures (brushing with a regular-headed mechanical or manual toothbrush, normal use of dental floss). Sutures were removed after 7–10 days. During the follow-up appointments at 2 and 4 weeks, gentle professional oral hygiene procedures were carried out by the clinician with extreme care not to injure the operated area. The guided biofilm technique (GBT) protocol with glycine powder was used to gently remove any biofilm in the operated area. During the follow-up appointments at 3, 6, 9, and 12 months postoperatively, professional oral hygiene procedures were carried out using the same GBT protocol, and OHI were reinforced.

### 2.9. Outcome Measures

Follow-up clinical parameters were recorded in both centers at 12 months post-surgery for six sites per tooth (mesio-buccal, mid-buccal, disto-buccal, mesio-oral, mid-oral, and disto-oral) using a manual periodontal probe (PCP-UNC 15^®^, Hu-Friedy, Chicago, IL, USA). Statistical analysis included measurements taken at baseline and 12 months after surgery only in the area of interest.

The primary outcome variable was CAL gain. The secondary outcome variables were PPD, GR, FMPS, and FMBS. For CAL, PPD, and GR, the tooth was considered as the statistical unit, while for FMPS and FMBS, the patient was considered as the statistical unit.

### 2.10. Statistical Analysis

Statistical analysis for our study was handled using Microsoft Excel (version 2019, Microsoft Corporation, Redmond, WA, USA), which facilitated the organized collection and preliminary analysis of study data. Statistical analyses were conducted using Python (version 3.8, Python Software Foundation, Wilmington, DE, USA) [38]. The Kolmogorov–Smirnov test was used to determine the normality of continuous variables including CAL, PPD, and GR. Normally distributed variables were described using means and standard deviations (SD) to capture the central tendency and dispersion of our data, while the mean differences captured between study groups were compared using independent sample t-tests. Categorical variables, such as demographic characteristics (sex distribution, age range, and smoking status), were summarized using frequencies and percentages, providing a clear overview of the study population’s composition. For categorical variables and proportions, the chi-square test was employed to examine the distribution differences between the groups. In instances where the assumptions for the chi-square test were not met, Fisher’s exact test was utilized as a reliable alternative. A *p*-value threshold of less than 0.05 was set for statistical significance across all tests.

## 3. Results

### 3.1. Study Population

At 12 months follow-up and completion of the study, data from 60 patients, 30 in each group (20 subjects from the University of Naples with 10 test and 10 control subjects and 40 subjects from the University of Timisoara with 20 test and 20 control subjects), was available for analysis. The study population consisted of 26 females and 34 males aged between 30 and 60 years. No intraoperative or postoperative complications of significance were observed in any of the patients.

The demographic characteristics analysis showed that sex distribution across the groups was closely matched, indicating no significant difference in gender distribution between the groups. The difference in mean age between the groups was also not statistically significant (*p* = 0.153), and further analysis showed a well-balanced age distribution. The prevalence of smoking, a potential confounder in periodontal studies, was also similar between the groups, with 26.7% smokers in the test group and 20.0% in the control group (*p* = 0.541), as presented in Table 1.

### 3.2. Changes in Clinical Attachment Level (CAL)

After 12 months, there was a statistically significant difference in both groups as compared to the baseline. The baseline intergroup comparison failed to show any statistically significant differences. However, follow-up intergroup measurements revealed statistically significant differences in the mean CAL gain (3.06 ± 1.13 mm vs. 1.44 ± 1.07 mm) with *p* < 0.001, indicating that the test group experienced significantly greater improvements in CAL gain than the control group (Table 2).

### 3.3. Changes in Pocket Probing Depth (PPD)

Baseline measurements of PPD between the groups revealed no statistically significant differences. However, after 12 months, the intragroup comparison in both groups revealed a reduction in PPD that was statistically significant (*p* < 0.0001). The intergroup comparison at 12 months revealed a mean PPD reduction of test group measurements (3.28 ± 1.14 mm) with respect to control group measurements (2.61 ± 1.22 mm) that was statistically significant *p* = 0.032 (Table 2).

### 3.4. Changes in the Gingival Recession (GR)

At 12 months, the intragroup comparison revealed minimal and not statistically significant changes in the GR in the test group. Conversely, the control group experienced statistically significant increases in GR (*p* < 0.001). The intergroup comparison at 12 months revealed statistically significant differences in the changes in mean GR that increased in the control group (*p* < 0.001), while in the test group, changes in GR were minimal and not statistically significant. The intergroup baseline comparison showed no statistically significant difference in the GR measurements (Table 2).

**Figure 3:**
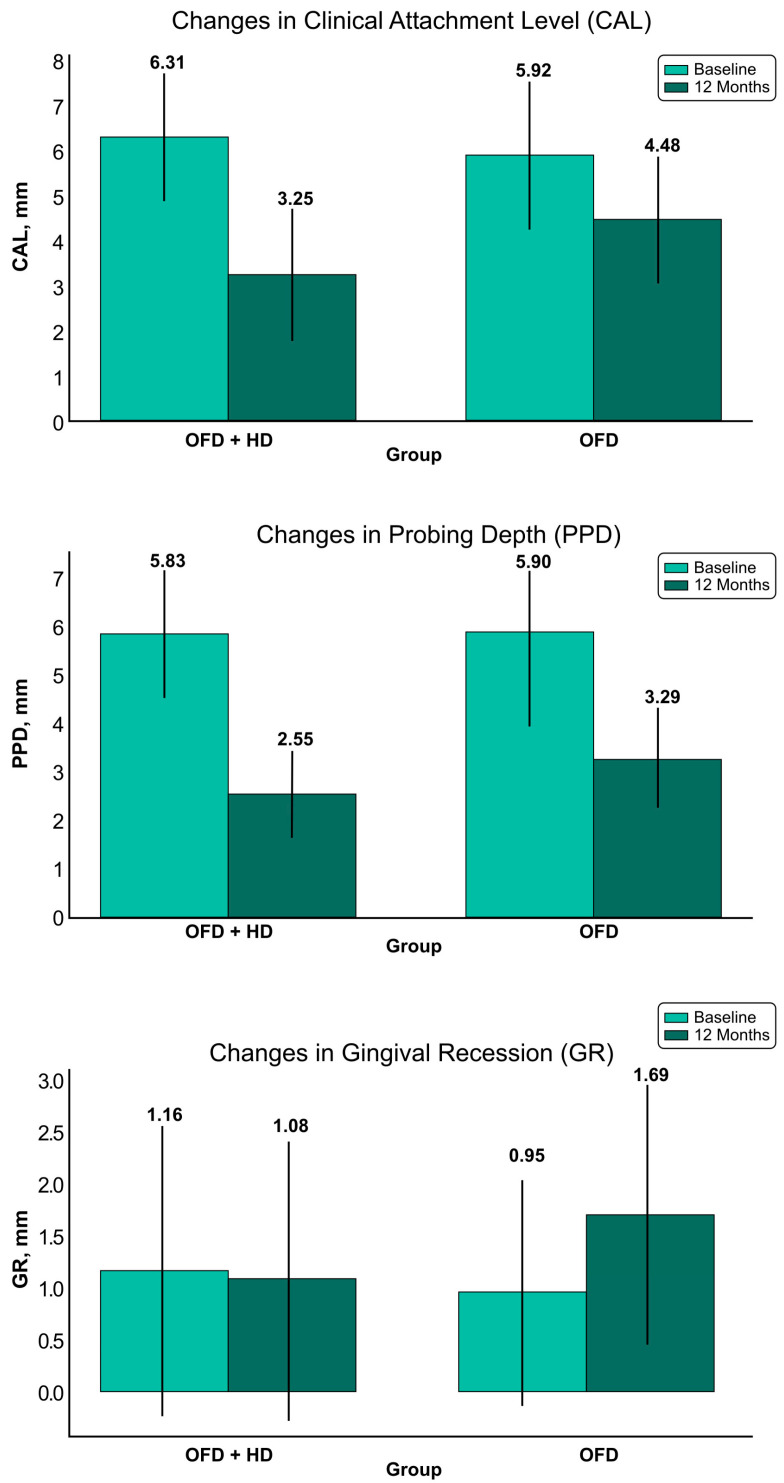
Graphic representation of clinical parameters changes. Abbreviations: OFD + HA—test group; OFD—control group.

The changes in clinically measured parameters (CAL, PPD, and GR) are presented in Figure 3.

### 3.5. Full-Mouth Plaque Score (FMPS) and Full-Mouth Bleeding Score (FMBS)

The intragroup comparison for the test group showed a reduction of FMPS at 12 months, resulting in a mean difference of 1.37 ± 1.69, which was statistically significant (*p* = 0.014). In contrast, the control group also showed improvement at the 12-month intragroup comparison, with a mean difference of 0.93 ± 1.05 but no statistical significance. The intergroup statistical analysis comparing the mean changes in FMPS between the two groups did not reveal a significant difference (*p* = 0.230) (Table 3).

In the test group, the intragroup comparison described a notable decrease of FMBS, with a mean difference of 2.50 ± 1.88, reflecting a statistically significant reduction in bleeding on probing (*p* < 0.001) at 12 months. Similarly, the control group’s FMBS scores also reflected a statistically significant reduction (*p* < 0.001), with a mean difference of 1.50 ± 1.42 at the 12 month intragroup comparison. While both treatments were effective in reducing gingival bleeding, the difference in mean changes of FMBS between the two treatments was statistically significant (*p* = 0.023) at the 12 month intergroup comparison, indicating that OFD + HA treatment was more effective than OFD only in reducing bleeding on probing, as described in Table 4.

### 3.6. Frequency Distributions of CAL, PD, and GR Changes

**Figure 4:**
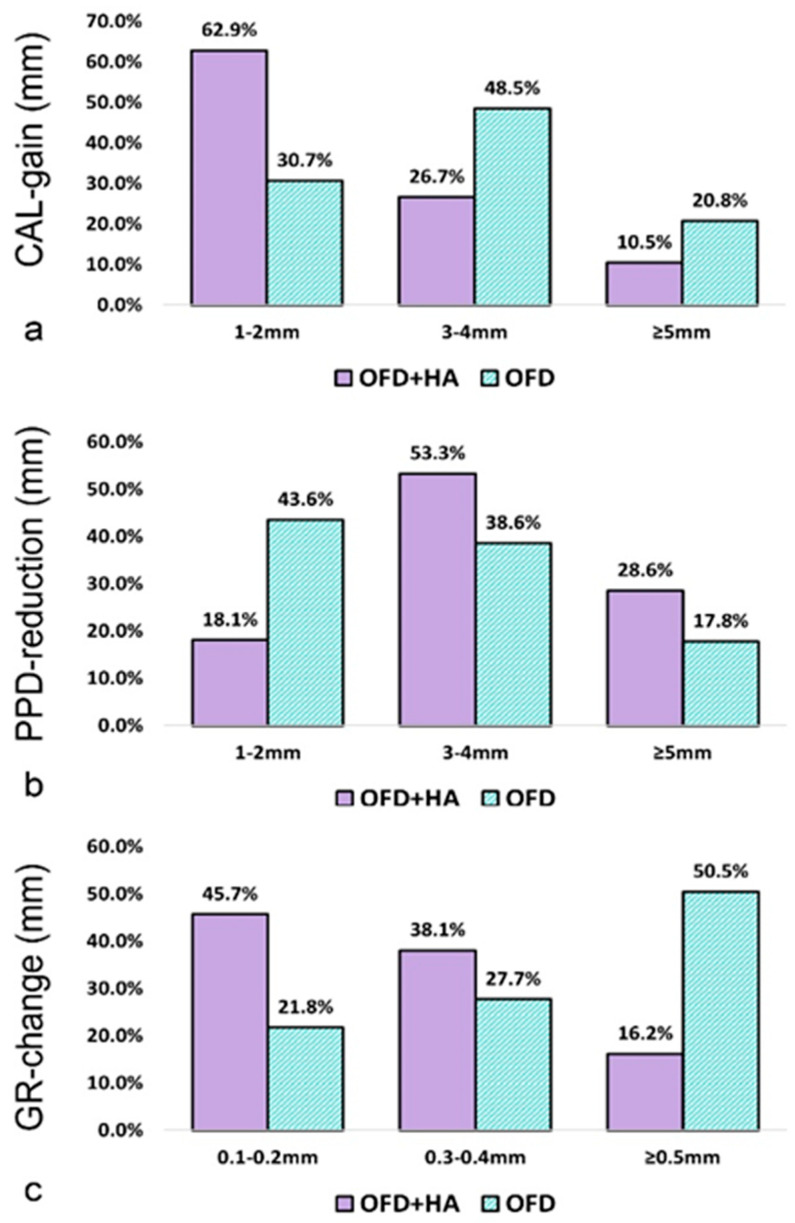
Frequency distribution of clinical parameter changes (expressed as % of sites) at 12 months: (**a**) CAL-gain, (**b**) PPD—reduction, (**c**) GR-change. Abbreviations: CAL—clinical attachment level; PPD—probing depth; GR—gingival recession; test group—OFD + HA; control group—OFD.

In the test group, 62.9% of sites experienced CAL gain of 1–2 mm, compared to 30.7% of sites in the control group. More than half of the test group sites exhibited a PPD reduction of 3–4 mm, and 28.6% reached a PPD reduction of ≥5 mm compared to the control group (17.8%). The GR change was more visible for the control group; in 50.5% of sites, the recession was ≥0.5 mm. Frequency distribution analysis is described in Figure 4.

## 4. Discussion

The present multicentric, randomized, controlled clinical trial has evaluated the clinical outcomes following treatment of suprabony periodontal defects with either OFD + HA or OFD only after an observation period of 12 months. After 12 months, the *p*-value of the mean CAL change was statistically significant (*p* < 0.001), therefore the null hypothesis was rejected. The hyaluronic acid application demonstrated its beneficial impact on cell types associated with soft and hard tissue regeneration in various preclinical studies [26,39,40]. Previous research examined the additional impact of hyaluronic acid (HA) administration in treating chronic periodontitis [41,42,43]. Its use in combination with periodontal surgery was believed to produce comparable positive outcomes. In the current study, HA gel was applied in conjunction with OFD. The surgical procedure aimed to evaluate the therapeutic effects of HA gel without removing the entire pocket lining, unlike other studies that used the modified Widman flap design [24]. To the best of our knowledge, no other comparative clinical research has been carried out using HA in the treatment of suprabony defects. Hence, the Discussion Section will refer to the same type of surgical treatment for suprabony defects in conjunction with another biomaterial (e.g., EMD).

The clinical significance of incorporating HA as an adjunctive biomaterial in periodontal surgical treatments for suprabony osseous defects lies in the user-friendly nature of the HA product (hyaDENT) and the absence of specific surgical requirements for achieving optimal outcomes, unlike other biomaterials, such as EMD, which necessitate specific surgical conditions for obtaining the best results.

The statistical analysis at 12 months revealed that both groups experienced a statistically significant CAL gain. These results are in accordance with those of recent clinical research that compared OFD with and without the additional application of EMD [6], thus suggesting that the additional application of HA could have similar results in terms of CAL gain as the additional application of enamel matrix proteins.

The intergroup comparison observed a CAL gain difference of 1.23 mm, which is statistically significant (*p* < 0.0001), favoring the test group. These findings are not in accordance with similar studies of suprabony defects treated with EMD, where the observed differences were 1.8 mm and 1.94 mm between test and control groups, respectively [36,44]. The surgical technique utilized (simplified papilla preservation flap [44]) could be one reason for these differences in CAL gain.

At 12 months, the PPD reduction was statistically significant in both groups. These results are in accordance with several studies that reported a significant PPD reduction compared to baseline for both test and control groups [36,44]. The difference between the test and control group revealed a mean reduction of 0.74 mm that is statistically significant (*p* < 0.0001) and in accordance with a previous recent study comparing the adjunctive effect of EMD with OFD in the treatment of suprabony defects [6]. This difference, however, is slightly lower when compared with results obtained by previous research that described 0.9 mm and 1.2 mm difference, respectively, favoring the test group (e.g., EMD) after 12 months [36,44]. Again, surgical protocol [36] and biomaterial [36,44] used are most likely to be the reasons for this discrepancy.

Mean GR changes of 0.74 mm (statistical significance, *p* < 0.0001) at 12 months were only observed in the control group at the intragroup comparison. These results are not in accordance with the 0.9 mm change reported in recent research that observed a statistically significant GR change in both groups at the 12 month intragroup comparison [6]. Again, it could be speculated that the biomaterial used in the previous study (e.g., EMD) may be the reason for this discrepancy. Another previous research speculated that EMD could generate a greater contraction in tissues compared to HA [30]. The intergroup comparison revealed a statistically significant change in the GR parameter, with a more pronounced GR change in the control group than in the test group, which is in accordance with several other studies that compared OFD with and without the adjunctive application of a biomaterial [36,44]. These findings underscore the complexity of managing gingival recession in periodontal therapy. While OFD alone appears to contribute to an increase in GR, the adjunctive use of HA seems to offer a stabilizing effect, limiting the progression of recession.

In our study, the FMPS reductions were statistically significant only for the test group at the 12 month intragroup comparison. Our results are in accordance with studies that used HA in conjunction with step 1 of periodontal therapy [23]. The comparison between the groups’ mean differences in FMPS did not reach statistical significance (*p* = 0.443), suggesting that both treatments were similarly effective in reducing plaque levels, most likely due to the recall protocol employed during the 12 month waiting period.

Although both groups saw improvements, the reduction in FMBS was statistically significant and more pronounced in the test group, suggesting that HA may have an additional beneficial effect on reducing gingival bleeding. This result reflects data resulting from a previous clinical and histological study that confirmed by gingival biopsy that HA significantly reduces the inflammatory infiltrate [45]. However, the intergroup difference in mean changes of FMBS approached, but did not reach, statistical significance (*p* = 0.055), indicating a trend toward greater improvement with HA but not conclusively, as described in Table 3.

During the entire period of the present study, none of the 60 patients from the two research centers reported any signs of complications. This is in accordance with previous research that used the same HA gel preparation, confirming once again the safety of hyaDent BG^®^ [24,28].

One limitation of the present study that could be mentioned is the lack of placebo gel application in the control group. A placebo gel was not used because an adequate placebo gel could not be obtained. Similar research that investigated the efficacy of HA in intrabony defects did not include a placebo group [24,46,47]. The lack of radiographic measurements could also be considered a limitation. However, prior similar research revealed that minimal bone gain could be anticipated in suprabony defect regeneration with biomaterials [44,48]. One year following treatment of suprabony defects with OFD + EMD, either a 0.26 mm [44] or a 0.10 mm [48] radiographic bone level gain was reported. Not including smoking patients or patients with systemic disorders could be considered another limitation of the study. However, being the first randomized clinical trial that describes the adjunctive effects of HA in the periodontal surgical treatment of suprabony osseous defects, it was considered that these risk factors (smoking and systemic disorders) could impair periodontal healing after surgery, thus masking the potential benefits of HA. Future research should focus on including smoking vs. nonsmoking groups and patients with systemic disorders vs. healthy patient groups for a better understanding of the beneficial effect that additional application of HA has in the surgical treatment of periodontal disease.

In summary, the results have shown that the additional application of HA to OFD yielded statistically significantly higher improvements in terms of CAL gain and PD reduction compared with treatment with OFD alone.

## 5. Conclusions

Within its limitations, the present research suggests that suprabony periodontal defects could benefit from the additional application of HA in conjunction with OFD in terms of improvement of all analyzed clinical parameters when compared with OFD alone. The null hypothesis was rejected. Horizontal bone defects could benefit as much as vertical intrabony defects from the adjunctive use of HA in surgical therapy of periodontitis.

## Figures and Tables

**Table 1:** Demographic characteristics.

Variable	Test Group (*n* = 30)	Control Group (*n* = 30)	*p*-Value
Gender (male/female)	18 (m); 12 (f)	16 (m); 14 (f)	0.602 ^a^
Age (mean ± SD)	49.8 ± 7.4	46.9 ± 8.1	0.153 ^b^
Tobacco smokers	8 (26.7%)	6 (20.0%)	0.541 ^a^

All data are expressed as mean and standard deviation. Abbreviations: SD—standard deviation; test group—OFD + HA treatment; control group—OFD treatment only; *n*—number of patients. Performed statistical tests: ^a^ Chi-square test; ^b^ Student’s *t*-test. Level of significance *p* < 0.05.

**Table 2:** Changes in CAL, PPD, and GR.

Variable (mm)	Test Group Baseline (*n* = 105)	Test Group 12 Months (*n* = 105)	Mean Difference (*p*-Value)	*p*-Value Baseline	Control Group Baseline (*n* = 101)	Control Group 12 Months (*n* = 101)	Mean Difference (*p*-Value)	*p*-Value 12 Months
CAL (mean ± SD)	6.31 ± 1.44	3.25 ± 1.50	3.06 ± 1.13 **(<0.0001)**	0.073	5.92 ± 1.66	4.48 ± 1.42	1.44 ± 1.07 **(<0.0001)**	**<0.001** ^a^
PPD (mean ± SD)	5.83 ± 1.31	2.55 ± 0.94	3.28 ± 1.14 **(<0.0001)**	0.699	5.90 ± 1.28	3.29 ± 1.05	2.61 ± 1.22 **(<0.0001)**	**0.032** ^a^
GR (mean ± SD)	1.16 ± 1.40	1.08 ± 1.36	0.08 ± 0.76 (=0.6749)	0.234	0.95 ± 1.10	1.69 ± 1.25	0.74 ± 1.03 **(<0.0001)**	**<0.001** ^a^

All data are expressed as mean and standard deviation. Performed statistical tests: ^a^ Student’s *t*-test; Abbreviations: CAL—clinical attachment level; PPD—probing depth; GR—gingival recession; SD—standard deviation; test group—OFD + HA treatment; control group—OFD treatment only; *n*—number of sites. Notes. *p*-values in bold indicate statistically significant differences (*p* < 0.05).

**Table 3:** Changes in FMPS and FMBS.

	Baseline	Baseline 95%CI	Follow-Up	Follow-Up 95%CI	Change	Change 95%CI	*p*-Value
**FMPS% (mean ± SD)**							
TEST	20.3 ± 2.0	19.5 to 22.2	19.0 ± 2.0	18.1 to 21.0	1.37 ± 1.69	0.5 to 1.6	**0.014** ^a^
CONTROL	20.2 ± 2.7	19.4 to 23.5	19.3 ± 2.0	18.5 to 21.9	0.93 ± 1.05	0.6 to 1.2	0.147 ^a^
*p*-value	0.871		0.563		0.230		
**FMBS% (mean ± SD)**							
TEST	18.9 ± 1.8	18.0 to 19.5	16.4 ± 2.1	15.2 to 18.0	2.50 ± 1.88	1.1 to 3.0	**<0.001** ^a^
CONTROL	19.2 ± 2.0	18.2 to 21.0	17.7 ± 2.4	16.3 to 19.3	1.50 ± 1.42	0.9 to 2.2	**<0.001** ^a^
*p*-value	0.543		**0.029**		**0.023**		

All data are expressed as mean and standard deviation. Abbreviations: FMPS—full-mouth plaque score; FMBS—full-mouth bleeding score; SD, standard deviation; test group—OFD + HA treatment; control group—OFD treatment only. Performed statistical tests: ^a^ Student’s *t*-test; Notes. *p*-values in bold indicate statistically significant differences (*p* < 0.05).

**Table 4:** Full-mouth plaque score (FMPS) and full-mouth bleeding score (FMBS).

Variable	Test Group Baseline (*n* = 30)	Test Group 12 Months (*n* = 30)	Mean Difference (*p*-Value)	*p*-Value Baseline	Control Group Baseline (*n* = 30)	Control Group 12 Months (*n* = 30)	Mean Difference (*p*-Value)	*p*-Value 12 Months
FMPS% (mean ± SD)	20.3 ± 2.0	19.0 ± 2.0	1.37 ± 1.69 **(0.014)**	0.871	20.2 ± 2.7	19.3 ± 2.0	0.93 ± 1.05 (0.147)	0.563 ^a^
FMPS range			0.043				0.116	0.443 ^b^
FMBS% (mean ± SD)	18.9 ± 1.8	16.4 ± 2.1	2.50 ± 1.88 **(<0.001)**		19.2 ± 2.0	17.7 ± 2.4	1.50 ± 1.42 **(0.010)**	**0.029** ^a^
FMBS range			0.003	0.544			0.307	0.055 ^b^

Abbreviations: FMPS—full-mouth plaque score; FMBS—full-mouth bleeding score; SD, standard deviation; test group—OFD + HA treatment; control group—OFD treatment only. Performed statistical tests: ^a^ Student’s *t*-test; ^b^ chi-square test; Notes. *p*-values in bold indicate statistically significant differences (*p* < 0.05).

## Data Availability

The data presented in this study are available upon request from the corresponding author.
